# Mitochondrial Genome Structures and Phylogenetic Analyses of Two Tropical Characidae Fishes

**DOI:** 10.3389/fgene.2021.627402

**Published:** 2021-02-01

**Authors:** Cheng-He Sun, Hong-Yi Liu, Nan Xu, Xiao-Li Zhang, Qun Zhang, Bo-Ping Han

**Affiliations:** ^1^Department of Ecology, Jinan University, Guangzhou, China; ^2^College of Biology and the Environment, Nanjing Forestry University, Nanjing, China

**Keywords:** Characidae, *Hemigrammus erythrozonus*, *Hyphessobrycon amandae*, mitochondrial genome, phylogeny

## Abstract

The Characidae family contains the largest number of tropical fish species. Morphological similarities make species identification difficult within this family. Here, the complete mitogenomes of two Characidae fish were determined and comparatively analyzed with those of nine other Characidae fish species. The two newly sequenced complete mitogenomes are circular DNA molecules with sizes of 16,701 bp (*Hyphessobrycon amandae*; MT484069) and 16,710 bp (*Hemigrammus erythrozonus*; MT484070); both have a highly conserved structure typical of Characidae, with the start codon ATN (ATG/ATT) and stop codon TAR (TAA/TAG) or an incomplete T−−/TA−. Most protein-coding genes of the 11 Characidae mitogenomes showed significant codon usage bias, and the protein-coding gene *cox1* was found to be a comparatively slow-evolving gene. Phylogenetic analyses via the maximum likelihood and Bayesian inference methods confirmed that *H. amandae* and *H. erythrozonus* belong to the family Characidae. In all Characidae species studied, one genus was well supported; whereas other two genera showed marked differentiation. These findings provide a phylogenetic basis for improved classification of the family Characidae. Determining the mitogenomes of *H. erythrozonus* and *H. amandae* improves our understanding of the phylogeny and evolution of fish species.

## Highlights

- The first complete mitogenomes of *Hemigrammus erythrozonus* and *Hyphessobrycon amandae* were assembled.
- The phylogenetic relationships among Characidae fishes were deduced using complete mitogenomes.
- These data are important for phylogenetic and taxonomic studies on Characidae.

## Introduction

The mitochondrion is an organelle that can directly convert organic matter into energy to support the biological activities of a cell (Avise et al., 1987; Wataru et al., 2013; Strohm et al., 2015; Parhi et al., 2019). It is the main site of ATP production and oxidative phosphorylation in eukaryotic cells (Wilson et al., 1985). Mitochondria possess mitochondrial DNA (mtDNA), which has a closed circular double-stranded structure and self-replicates semi-conservatively (Prosdocimi et al., 2012; Paz et al., 2014). Mitochondrial DNA is considered the second genetic information system of eukaryotic cells (Kim et al., 2008; Cooke et al., 2012; Zhao et al., 2015; Ruan et al., 2020). Compared with nuclear DNA, it is a relatively independent replication unit characterized by a small size, simple structure, maternal inheritance, rapid evolution, limited recombination, and variability in evolutionary rate at different loci (Harrison, 1989; Javonillo et al., 2010). These characteristics make mitochondrial genome (mitogenome) a valuable resource for studying DNA structure and gene expression, as well as for understanding the evolution and phylogenetic distribution of species.

Recent advances in molecular biology, such as second-generation sequencing technologies, have facilitated the sequencing of fish mtDNA, thereby providing a clearer understanding of the structures of fish mitogenomes, which are between 16 and 18 kb. Structurally, protein coding (PCG), transfer RNA (tRNA), and ribosomal RNA (rRNA) genes as well as the noncoding regions of fish mitogenomes are highly conserved, but gene intervals and lengths vary between species (Gray, 1989; Kim et al., 2009; Alam et al., 2014). As mtDNA sequences of many fish species have been determined, mitogenomes have become popular molecular guides in phylogenetic and evolutionary studies of fishes (Brown et al., 1979; Wang et al., 2016; Wu et al., 2020).

The Characidae family contains the largest number of species among tropical fishes and belongs to class Actinopterygii and order Characiformes (Wilkens, 1988; Mirande, 2010). This family is mainly found in the freshwater rivers and lakes of Africa and America and inhabits habitats with slow water flows. The fish species of this family are characterized by a small adipose fin on the caudal stalk. Most Characidae are small and harmless; only a few species are predatory. Because of the small body size and colorful markings, Characidae is the most popular tropical fish family raised. With the rapid development of the global trade market of ornamental fish, increase in fishing activities, and deterioration of ecological habitats, the natural resources that Characidae fish depend on have been seriously damaged. Accurate species identification and understanding of the systematic relationships among species are useful for protecting existing species and discovering new species. However, the classification of Characidae species remains difficult because of the morphological similarities among many species (Wilkens, 1971; Langerhans et al., 2003; Oliveira et al., 2011; Barreto et al., 2017).

As there are many types of ornamental fish available in the market and hybrid species are widespread, two common fishes were selected for this study. The main reason for choosing Glowlight tetras (*Hemigrammus erythrozonus*) and Ember tetra (*Hyphessobrycon amandae*) is that these two fishes are representative and common in the family Characidae, and their morphological characterization is relatively accurate. In this study, the complete mitochondrial genomes of two tropical fishes were sequenced, assembled, and annotated. The genome organization, gene contents, repeat sequences, and tRNA structures of the two newly sequenced mitogenomes were compared and analyzed. The mitogenomes of these two fishes were compared with those of nine other Characidae species to identify the similarities and differences in their gene orders, genetic structures, base compositions, evolutionary features, and codon usage. Additionally, phylogenetic analysis of various Characiformes species was carried out using a combined mitochondrial gene set. The mitogenomes of the two Characidae species improve our phylogenetic and evolutionary understanding of Characidae fishes.

## Materials and Methods

### Samples and DNA Extraction

The two specimens were collected from the Nanjing Qiqiaoweng flower and bird market, Jiangsu province, China (32°0′27.1′′N, 118°50′11.5′′E). Morphological identification was conducted during the sampling according to the latest taxonomic classification of fish. As these two species were collected from an ornamental fish market, the geographic data about the specific origins of the species are unknown. Total genomic DNA from the samples was extracted using a FastPure Cell/Tissue DNA Isolation Mini Kit V7.1 (Vazyme Biotech Co., Ltd., Nanjing, China) (Chen et al., 2018). DNA integrity was evaluated via 1.5% agarose gel electrophoresis. DNA concentration and purity were assessed using a NanoDrop 2000 (NanoDrop Technologies, Wilmington, NC, United States).

### PCR Amplification and DNA Sequencing

To amplify the mitogenomes of *Hemigrammus erythrozonus* and *Hyphessobrycon amandae*, nine pairs of specificity primers (Table 1) were designed based on the published conserved nucleotide sequences of nine Characidae mitogenomes (*Astyanax giton*, *Astyanax paranae*, *Gephyrocharax atracaudatus*, *Grundulus bogotensis*, *Hasemania nana*, *Hemigrammus bleheri*, *Oligosarcus argenteus*, *Paracheirodon axelrodi*, and *Paracheirodon innesi*). For accurate sequencing and assembly of the complete mitogenomes, the overlap between adjacent fragments was designed to exceed 200–300 base pairs (bp). Because of the differences in the mitogenomes between the two species, specific primers were designed. PCR amplification was performed as described previously (Sun et al., 2019a). The PCR products were electrophoretically separated on a 1.5% agarose gel and subsequently purified and Sanger-sequenced by Tsingke Biotech (Tsingke Biotechnology Co., Ltd., Nanjing, China).

**TABLE 1:** Primers for the PCR-amplification of the two Characidae mitogenome sequences.

**Primers**	**Nucleotide sequence (5′–3′)**	**Length (bp)**	**Gene or region**
GSY-F1	GCTTAACTAAAGCATAACGCTG	1,995	trnF-rrnL
GSY-R1	AAAGACAAGTGATTGCGCTACC		
GSY-F2	TCGTTAACCCCACACCGG	2,300	rrnL-nad2
GSY-R2	GCTTCTGTTGCTCGGGGGTGGTG		
GSY-F3	TAGAACTCAAAATTCTAAGT	2,110	nad2-cox1
GSY-R3	CACCACCCCCGAGCAACAGAAGC		
GSY-F4	CTGTCTACCCCCCTCTTGC	2,380	cox1-atp6
GSY-R4	CTGCCCTTGAAGTGTAAT		
GSY-F5	GAGACTGAACATGGCACTAGG	2,010	atp6-nad4l
GSY-R5	TAAATGACATAAGGGGGCTAT		
GSY-F6	GAATAAGGGGCTAGTCCAAC	2,210	nad4l-nad5
GSY-R6	AATTTAAAGCTGATGTTGATG		
GSY-F7	AACCCCGTACCCCAAAAACC	2,140	nad5-nad6
GSY-R7	GTGGTATTTTAGTGGGACATGG		
GSY-F8	CCCATATCACCCCTCATGAAGT	2,080	nad6-trnP
GSY-R8	TCAATTTACCCGTGAGGGGC		
GSY-F9	CTCAACCCACTAGCCGGCTT	1,806	trnP-trnF
GSY-R9	AGCTGATAGTAAAGTCGGGACC		

### Genome Assembly and Annotation

DNA sequencing results were verified using NCBI BLAST (Johnson et al., 2008). Raw sequence data from the DNA fragments were screened and assembled using Lasergene 7.1 (DNAStar, Inc. Madison, WI, United States) to obtain the complete mitogenome sequences. The tRNAscan-SE v2.0 (Lowe and Chan, 2016) software and MITOS WebServer^1^ (Bernt et al., 2013) were used to verify the tRNA genes. Open reading frame finder (Master et al., 2016; Sun et al., 2019b) and the NCBI website were used to identify the protein-coding regions by using the default settings for the vertebrate mitochondrial code, and GenBank was used to translate the putative proteins. The sequences of the identified PCGs and rRNAs were analyzed and compared with those of other Characidae species.

MEGA version 7.0 (Kumar et al., 2016) was used to determine base compositions, genetic distances, and relative synonymous codon usage values. The formula “AT-skew = (A − T)/(A ++ T)” (Perna and Kocher, 1995) was used to analyze strand asymmetry. DnaSP 5.1 (Librado and Rozas, 2009) was used to determine the rates of non-synonymous (Ka) and synonymous substitutions (Ks) and the ratio of Ka/Ks for the 13 Characidae species. The online software Ogdraw^2^ (Lohse et al., 2013) was used to generate circular mitogenome maps.

### Phylogenetic Analyses

To investigate the phylogenetic relationship between the two Characidae species, a phylogenetic tree of 24 Actinopterygii species (Table 2) was constructed based on the combined mitochondrial gene set (13 PCGs + two rRNAs). MAFFT v7.313 (Katoh and Standley, 2013) was used to perform multiple-sequence alignment. The maximum likelihood (ML) and Bayesian inference (BI) methods were used for phylogenetic analysis. ModelFinder (Kalyaanamoorthy et al., 2017) was used to select the best-fit substitution model and best partitioning scheme, a greedy algorithm was adopted with the Akaike information criterion (Yamaoka et al., 1978). ML method was used to construct an evolutionary tree by using IQ-TREE v.1.6.8 (Nguyen et al., 2015) based on the GTR + R + F model. The BI method was used to construct an evolutionary tree by using MrBayes v3.2.6 (Ronquist et al., 2012) based on the GTR + I + G + F model. Two independent runs with four chains each were simultaneously conducted for ten million generations, with one tree sampled every 100 generations. The first 25% of the samples was discarded as burn-in, and the remaining trees were used to calculate the Bayesian posterior probabilities. FigTree v1.4.0 (Rambaut, 2015) was used to visualize and edit the resulting phylogenetic evolutionary trees.

**TABLE 2:** List of the mitogenomes analyzed in this study.

**Order**	**Family**	**Species (Latin names)**	**Common name**	**GenBank no.**	**Size (bp)**
Characiformes	Acestrorhynchidae	*Acestrorhynchus* sp.		AP011981	16, 758
	Alestiidae	*Phenacogrammus interruptus*	Congo tetra	AB054129	16, 652
	Anostomidae	*Leporinus elongatus*	Piau verdadeiro	KU980144	16, 774
	Bryconidae	*Salminus brasiliensis*	Dorado	KM245047	17, 721
	Citharinidae	*Citharinus congicus*	Congo citharin	AP011985	16, 453
	Curimatidae	*Curimata mivartii*	Barbitetra	KP025764	16, 705
	Erythrinidae	*Hoplias intermedius*	Trairão	KU523584	16, 629
	Gasteropelecidae	*Carnegiella strigata*	Marbled hatchetfish	AP011983	17, 852
	Hemiodontidae	*Hemiodopsis gracilis*	Charuto	AP011990	16, 731
	Hepsetidae	*Hepsetus odoe*	Pike characid	AP011991	16, 803
	Lebiasinidae	*Lebiasina astrigata*	Guaija	MH921292	16, 899
	Serrasalmidae	*Piaractus brachypomus*	Pirapatinga	KJ993871	16, 722
	Characidae	*Astyanax giton*	Piaba do Rio Paraíba	MF805815	16, 643
		*Astyanax paranae*	Alambari	KX609386	16, 707
		*Gephyrocharax atracaudatus*	Platinunm tetra	MH636341	17, 049
		*Grundulus bogotensis*	Guapucha	KM677190	17, 123
		*Hasemania nana*	Silver-tipped tetra	AB861475	16, 581
		*Hemigrammus bleheri*	Red-nose tetra	LC074360	17, 021
		*Hemigrammus erythrozonus*	Glowlight tetra	MT484070	16, 710
		*Hyphessobrycon amandae*	Ember tetra	MT484069	16, 701
		*Oligosarcus argenteus*	Lambari-bocarra	MF805814	16, 711
		*Paracheirodon axelrodi*	Cardinal tetra	MH998225	17, 100
		*Paracheirodon innesi*	Neon tetra	KT783482	16, 962
Perciformes	Moronidae	*Lateolabrax japonicus*	Japanese seaperch	AP006789	16, 593

## Results and Discussion

### General Features of the Two Mitogenomes

**FIGURE 1:**
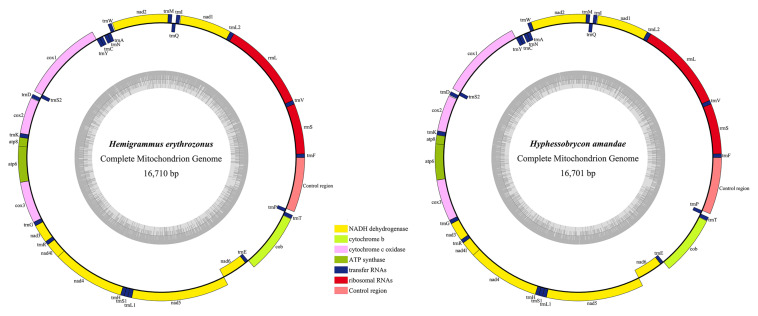
Gene maps of the two newly sequenced Characidae species. Genes encoded by the H-strand were showed outside the circle, and those encoded by the L-strand were showed inside the circle. Different gene types are shown as filled boxes in different colors. The gray inner circles showed the GC content in the mitogenome.

The two mitogenomes were found to be circular DNA molecules. The sizes of *H. erythrozonus* and *H. amandae* mitogenomes were 16,710 and 16,701 bp, respectively, (Table 3 and Figure 1), which are similar to the mitogenome sizes of other Characidae species, (Javonillo et al., 2010) such as *H. bleheri* (17,021 bp) and *O. argenteus* (16,711 bp). The gene arrangement and content of the two Characidae mitogenomes were typical of Characidae and highly conserved (Table 4), comprising 37 mitochondrial genes (13 PCGs, 22 tRNAs, and 2 rRNAs) and one control regions (CR). Eight tRNAs (*trnA*, *trnC*, *trnE*, *trnN*, *trnP*, *trnQ*, *trnS2*, and *trnY*) and *nad6* were found to be encoded on the L-strand (Figure 1), whereas 14 tRNAs, 12 PCGs, 2 rRNAs, and 1 CR were on the H-strand. Similar to other fish mitogenomes (Kim et al., 2008; Ruan et al., 2020), the A + T content in Characidae mitogenomes was highly biased, ranging from 57.1 (*A. paranae*) to 60.1% (*G. bogotensis*) (Table 4). The base composition of a mitogenome is frequently described in terms of the AT skew. The negligible A skew (0.020 and 0.016 for *H. erythrozonus* and *H. amandae*, respectively) in each sequenced mitogenome was similar to those in other Characidae and most fish species (Calcagnotto et al., 2005; Zhang et al., 2016).

**TABLE 3:** Gene annotations of the complete mitogenomes of *H. erythrozonus* and *H. amandae.*

**Gene**	**Strand**	**Position**		**Length (bp)**	**Start codons**	**Stop codons**	**Anticodon**	**Intergenic nucleotides**
		**From**	**To**					
trnF	H	1/1	68/69	68/69			GAA	0/0
rrnS	H	69/70	1017/1018	949/949				0/0
trnV	H	1018/1019	1089/1090	72/72			TAC	0/0
rrnL	H	1090/1091	2767/2761	1678/1671				0/0
trnL2	H	2768/2762	2842/2836	75/75			TAA	0/0
nad1	H	2843/2837	3814/3808	972/972	ATG/ATG	TAA/TAA		12/10
trnI	H	3827/3819	3898/3890	72/72			GAT	−2/−2
trnQ	L	3897/3889	3967/3959	71/71			TTG	11/−5
trnM	H	3979/3955	4051/4025	73/71			CGT	1/1
nad2	H	4053/4027	5111/5070	1059/1044	ATG/ATT	TAA/TAA		16/−1
trnW	H	5128/5070	5198/5140	71/71			TCA	6/6
trnA	L	5205/5147	5273/5215	69/69			TGC	1/1
trnN	L	5275/5217	5347/5289	73/73			GTT	31/30
trnC	L	5379/5320	5444/5385	66/66			GCA	−1/−1
trnY	L	5444/5385	5514/5455	71/71			GTA	1/1
cox1	H	5516/5457	7075/7016	1560/1560	GTG/GTG	AGG/AGG		−13/−13
trnS2	L	7063/7004	7134/7075	72/72			TGA	3/3
trnD	H	7138/7079	7209/7150	72/72			GTC	6/13
cox2	H	7216/7164	7903/7851	688/688	ATG/ATG	T/T		0/2
trnK	H	7904/7854	7976/7928	73/75			TTT	1/2
atp8	H	7978/7931	8145/8098	168/168	ATG/ATG	TAG/TAG		−10/−10
atp6	H	8136/8089	8818/8771	683/683	ATG/ATG	TA/TA		−1/−1
cox3	H	8818/8771	9601/9555	784/785	ATG/ATG	T/TA		0/−1
trnG	H	9602/9555	9674/9626	73/72			TCC	0/0
nad3	H	9675/9627	10025/9977	351/351	ATG/ATG	TAA/TAA		−2/−2
trnR	H	10024/9976	10092/10044	69/69			TCG	0/0
nad4l	H	10093/10045	10389/10341	297/297	ATG/GTG	TAA/TAA		−7/−7
nad4	H	10383/10335	11763/11715	1381/1381	ATG/ATG	T/T/		0/0
trnH	H	11764/11716	11832/11784	69/69			GTG	0/0
trnS1	H	11833/11785	11900/11852	68/68			GCT	1/1
trnL1	H	11902/11854	11974/11926	73/73			TAG	0/0
nad5	H	11975/11927	13813/13765	1839/1839	ATG/ATG	TAA/TAA		−4/−4
nad6	L	13810/13762	14325/14277	516/516	ATG/ATG	TAA/TAA		0/0
trnE	L	14326/14278	14393/14345	68/68			TTC	3/2
cob	H	14397/14348	15533/15484	1137/1137	ATG/ATG	TAA/TAA		4/4
trnT	H	15538/15489	15609/15559	72/71			TGT	−2/−2
trnP	L	15608/15558	15677/15627	70/70			TGG	0/0
Control region		15678/15628	16710/16701	1033/1074				0/0

**TABLE 4:** Base compositions of the whole genomes, protein-coding genes (PCGs), rRNAs, tRNAs, and Control regions of the 11 Characidae mitogenomes.

**Species**	**Whole genome**			**PCGs**			**rRNAs**			**tRNAs**			**Control region**		
	**Size (bp)**	**A+T (%)**	**AT− skew**	**Size (bp)**	**A+T (%)**	**AT− skew**	**Size (bp)**	**A+T (%)**	**AT−skew**	**Size (bp)**	**A+T (%)**	**AT−skew**	**Size (bp)**	**A+T (%)**	**ATskew**
*A. giton*	16,643	59.2	0.003	11,426	59.8	−0.087	2,618	55.6	0.201	1,558	58.0	0.017	977	64.3	0.089
*A. paranae*	16,707	57.1	0.033	11,432	57.1	−0.054	2,619	55.1	0.220	1,559	56.2	0.103	1,032	63.9	0.058
*G. atracaudatus*	17,049	58.5	0.022	11,430	58.3	−0.046	2,634	56.1	0.209	1,569	56.3	0.083	1,397	68.4	–0.020
*G. bogotensis*	17,123	60.1	0.008	11,421	60.2	−0.083	2,626	56.6	0.187	1,564	57.3	0.086	933	72.6	0.094
*H. amandae*	16,701	57.2	0.016	11,421	56.9	−0.065	2,620	56.3	0.200	1,559	55.9	0.034	1,074	65.2	–0.026
*H. bleheri*	17,021	58.4	0.003	11,424	57.9	−0.095	2,605	57.2	0.220	1,557	58.7	0.090	1,308	65.2	0.009
*H. erythrozonus*	16,710	57.5	0.020	11,435	57.4	−0.056	2,628	56.0	0.196	1,560	56.7	0.023	1,033	65.2	–0.015
*H. nana*	16,581	58.3	0.033	11,430	58.2	−0.045	2,621	56.0	0.221	1,556	58.3	0.070	933	65.5	0.011
*O. argenteus*	16,711	57.6	0.028	11,432	57.6	−0.063	2,618	55.7	0.214	1,559	56.0	0.014	1,037	64.6	0.071
*P. axelrodi*	17,100	59.0	0.003	11,184	58.7	−0.083	2,617	55.3	0.212	1,552	59.4	0.084	1,433	68.0	–0.015
*P. innesi*	16,962	58.5	0.012	11,429	58.5	−0.080	2,612	55.1	0.230	1,550	58.2	0.041	1,305	65.3	0.023

### Protein-Coding Genes

The total length of the PCGs in each of the 11 Characidae species ranged from 11,184 bp (*P. axelrodi*) to 11,435 (*H. erythrozonus*) (Table 4). Among these 11 sequenced mitogenomes, one PCG (*nad6*) was encoded on the L-strand, whereas the remaining PCGs were located on the H-strand. The average A + T content of the PCGs in each of the 11 Characidae species varied from 56.9 (*H. amandae*) to 60.2% (*G. bogotensis*). Most PCGs used the conventional start codon ATN (ATG/ATT), except for *H. erythrozonus cox1*, which started with GTG. Within our two newly sequenced mitogenomes, only the *cox1* and *nad4L* genes of *H. amandae* started with GTG (Table 3). Most PCGs terminated with the codon TAR (TAA/TAG) or incomplete codon (TA−/T−−), except for the *cox1* gene, which terminated with AGG, in both mitogenomes. As with Characidae mitogenomes, incomplete stop codons are commonly observed across fish mitogenomes (Cooper et al., 2001; Zhao et al., 2015), which may be related to post-transcriptional modification during mRNA maturation. The AT-skews (−0.095 to −0.045) of PCGs were similar among the 11 Characidae species (Table 4).

**FIGURE 2:**
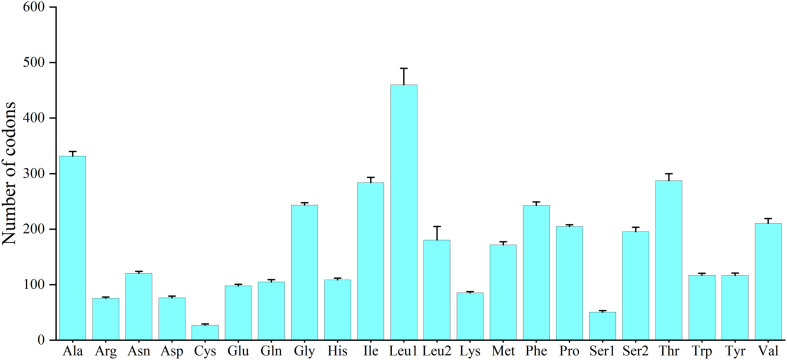
Codon usage patterns of 11 Characidae mitogenomes.

**FIGURE 3:**
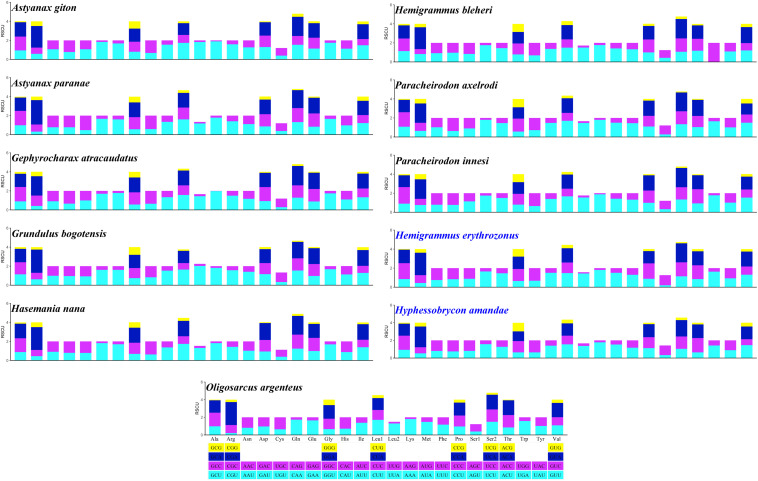
Relative synonymous codon usage of 13 protein-coding genes in the mitogenomes of 11 Characidae species. The codon families are shown on the *X*-axis, and the RSCU values are shown on the *Y*-axis. The different colors of the bars corresponds to the codon distribution at the bottom.

Excluding the stop codons, the mitogenome PCGs consisted of 3,718–3,801 codons (CDs) and showed very similar codon usage among the 11 Characidae species (Figure 2). Ile (283.64 ± 9.67 CDs), Thr (287.36 ± 12.52 CDs), Ala (331.36 ± 8.67 CDs), and Leu1 (CUN) (459.91 ± 29.62 CDs) were the four most predominant codon families. Among these, Leu1 (CUN) exhibited the highest usage bias (402–508 CDs), which may be associated with the coding function of the chondriosome. In contrast, Cys (27.27 ± 1.81 CDs) showed the least number of CDs. To gain an insight into the genetic codon bias of the 11 Characidae mitogenomes, the relative synonymous codon usage was evaluated. As shown in Figure 3, the usage of synonymous codons was biased for most amino acids. Moreover, the synonymous codon preferences for the 11 Characidae species were conserved, which may be attributed to their close relationship in the same fish family; these preferences have also been observed in some other fishes (Parhi et al., 2019). The two most commonly used codons in these 11 species were consistently AUU and CUU.

**FIGURE 4:**
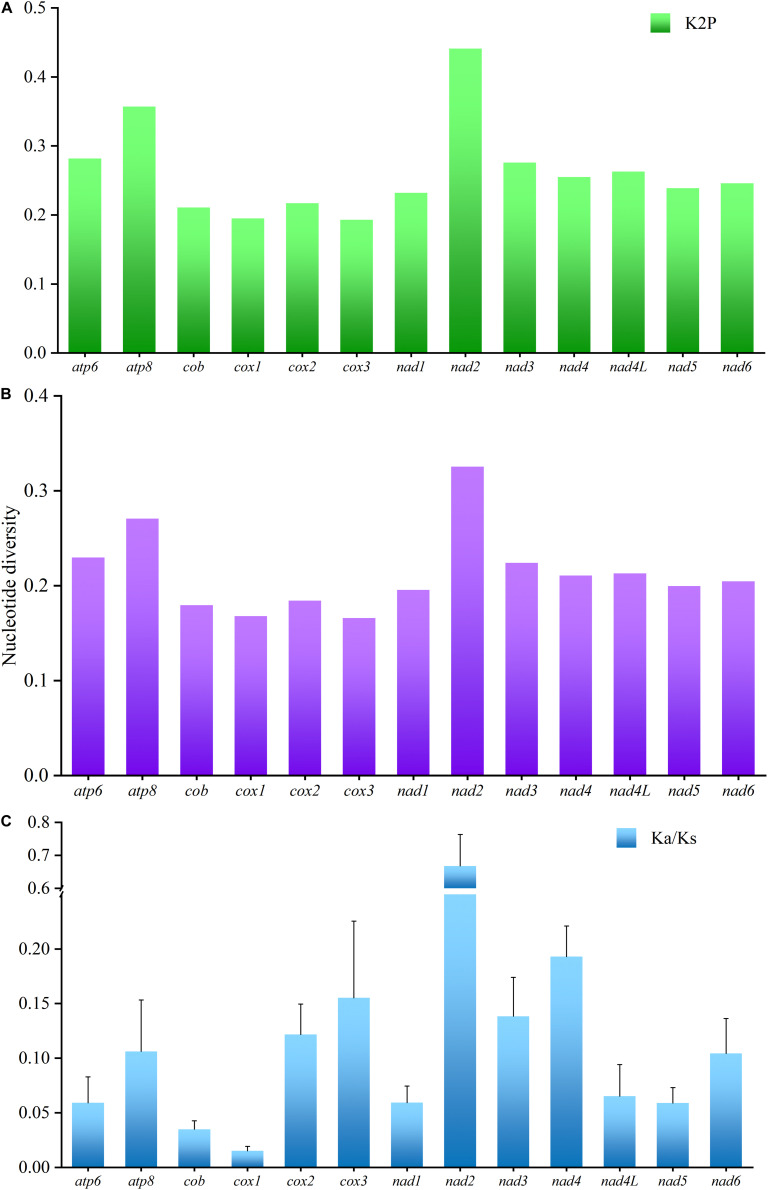
K2P genetic distance **(A)**, nucleotide diversity **(B)**, and the Ka/Ks ratio **(C)** analyses of the protein-coding genes among 11 Characidae mitogenomes.

To analyze the evolutionary pattern of the PCGs, the ratio of Ka/Ks, nucleotide diversity, and K2P genetic distance across all Characidae mitogenomes were calculated for each aligned PCG. Among the PCGs detected, *nad2* showed the largest K2P genetic distance among the 11 Characidae species (Figure 4A), followed by *atp8* and *atp6*. As seen in Figure 4B, *nad2* and *atp8* had the highest nucleotide diversity; in contrast, *cox1* and *cox3* had the lowest nucleotide diversity. Similar to the nucleotide diversity, Ka/Ks value was the highest for *nad2*, followed by *nad4*, *cox3*, and *nad3*; the lowest value was observed for *cox1* and *cob* (Figure 4C). Notably, the Ka/Ks values were <1 in all the PCGs, suggesting that all the PCGs have evolved under purifying selection. Based on the above-mentioned analyses, *nad2* is the most rapidly evolving gene among Characidae mitochondrial PCGs, since it is under the least selection pressure. In contrast, *cox1* is the most slowly evolving gene due to the highest selection pressure it is subjected to.

### Ribosomal and Transfer RNA Genes

The sizes of the 16S rRNA genes were 1,678 bp (*H. erythrozonus*) and 1,671 bp (*H. amandae*), and the 12S rRNA genes of both mitogenomes were 949 bp. The rRNA genes of Characidae mitogenomes were found to be highly conserved compared with those of other published fish mitogenomes (Javonillo et al., 2010; Zhao et al., 2015; Ruan et al., 2020), with the two rRNA genes located between *trnL2* and *trnF* separated by *trnV*. The A + T contents of rRNA genes ranged from 55.1 to 57.2% among the 11 Characidae species (Table 4). For the two newly sequenced Characidae mitogenomes, the typical 22 tRNAs were detected. Among them, 14 tRNAs were encoded on the H-strand, and the remaining eight on the L-strand.

**FIGURE 5:**
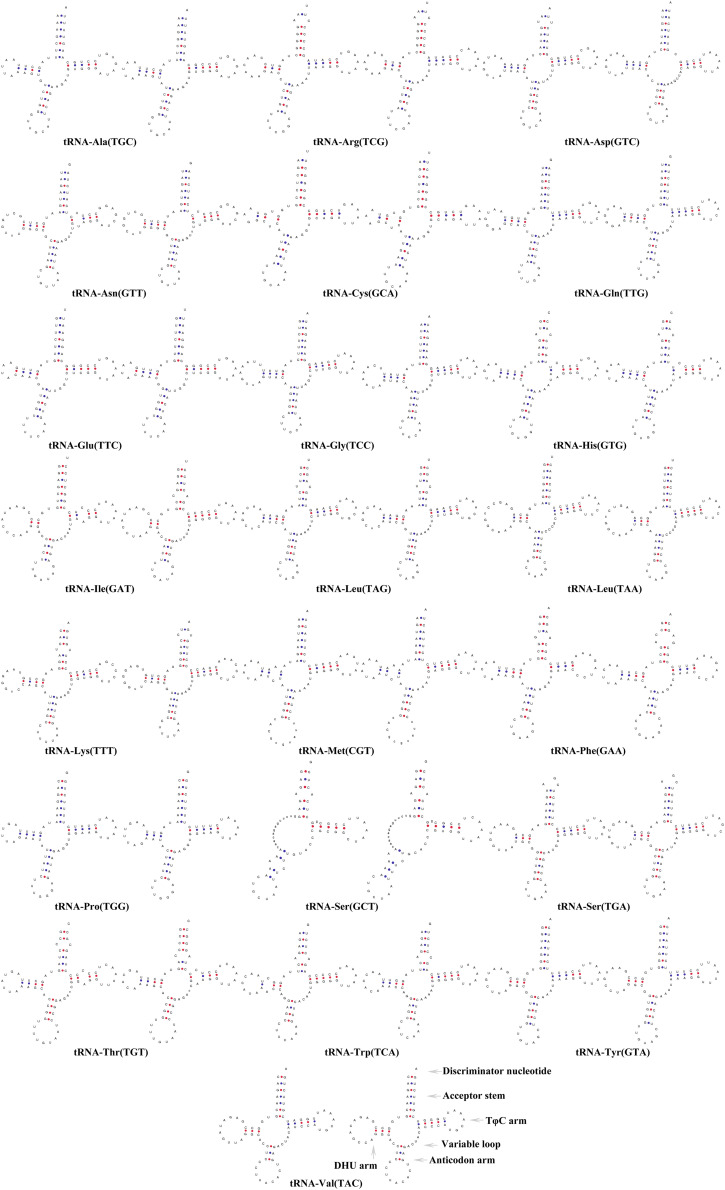
Secondary structures of the 22 transfer RNA genes of two Characidae species [*Hemigrammus erythrozonus* (left) and *Hyphessobrycon amandae* (right)].

The sizes of the tRNA genes ranged from 66 bp (*trnC*) to 75 bp (*trnL2*) in both *H. erythrozonus* and *H. amandae.* The total lengths of the 22 tRNA genes ranged from 1,550 bp (*P. innesi*) to 1,529 bp (*G. atracaudatus*) among the 11 Characidae. As shown in Figure 5, all the tRNAs exhibited a typical clover-leaf secondary structure, except for *trnS1* (GCT), which lacked the dihydrouridine arm, a feature generally present in Characidae fishes and vertebrate mitogenomes (Krajewski et al., 2010; Sun et al., 2020a,b).

### Control Region

Compared with PCGs and rRNA genes, the CR displayed the highest variation and mutation rates throughout the mitogenomes; thus, this region was the dominant region for evaluating intraspecies variations. The CR has become a hotspot for phylogenetic research since this region shows the maximum mutation and fastest evolution rates in the whole mitogenomes. Similar to other fish mitogenomes, the CRs were found to be located between *trnF* and *trnP* in all the 11 Characidae species. The average A + T content (63.9–72.6%) of the CRs was higher than that of the whole genomes (57.1–60.1%), PCGs (56.9–60.2%), rRNAs (55.1–57.2%), or tRNAs (55.9–59.4%). Composition analysis revealed seven positive and four negative AT skew regions in the mitogenome CRs of the 11 Characidae species.

### Phylogenetic Analyses

**FIGURE 6:**
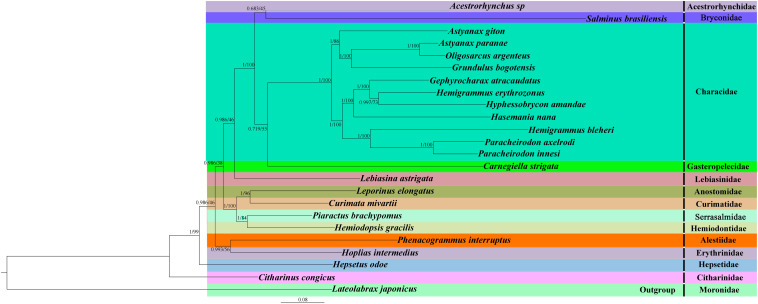
Phylogenetic tree of 24 Actinopterygii species constructed by the Bayesian inference and maximum likelihood methods based on the concatenated sequences of 13 PCGs and two rRNAs. The support values are Bayesian posterior probabilities (left) and bootstrap support values (right), respectively.

To determine the phylogenetic relationship between *H. erythrozonus* and *H. amandae* in the family Characidae, we selected the concatenated nucleotide sequences of the combined mitochondrial gene set (13 PCGs + two rRNAs) from 23 Characiformes species. Additionally, we used *Lateolabrax japonicas* (Lavoue et al., 2014) as an outgroup because it belongs to the order Perciformes and family *Moronidae*. As shown in Figure 6 and Supplementary Figures 1, 2, the phylogenetic analysis of the two tree models (BI and ML) by using the combined mitochondrial gene set well supported the tree topologies and yielded identical results. All the major clades were supported in the preferred trees by the analysis.

Although the experimental samples were from an animal market and there is a lack of comparison among wild samples, mitochondria are inherited from the maternal line, and we have a good morphological classification basis. Therefore, we believe that, even if samples are gathered from an animal market, the corresponding results will not be compromised by analysis bias as long as the morphological identification is performed well.

Two target species, *H. erythrozonus* and *H. amandae*, and nine other Characidae were clustered into one branch with a high nodal support value (BI posterior probabilities [PP] > 0.99; ML bootstrap [BP] > 70). This result confirmed the classification statuses of *H. erythrozonus* and *H. amandae* in Characidae. In line with previous reports (Mirande, 2019; Montero-Mendieta and Dheer, 2019), our study proves that *P. brachypomus* and *S. brasiliensis* do not belong to the family Characidae. *Piaractus* is a member of Serrasalmidae, and *Salminus* is a member of Bryconidae. *A. paranae* and *O. argenteus* form a well-supported clade. Likewise, *P. axelrodi* and *P. innesi* form a separate well-supported clade (PP = 1; BP = 100). In all the Characidae species studied, one genus was well supported (*P. axelrodi* and *P. innesi*), and the other two genera diverged (*A. giton* and *A. paranae*, and *H. erythrozonus* and *H. bleheri*). This two genera have been discussed in a recent taxonomic study. The taxonomic status of three species has been reassessed: *Hemigrammus bleheri* should be *Petitella bleheri* (Bittencourt et al., 2020), renamed *Astyanax giton* as *Deuterodon giton*, and *Astyanax paranae* as *Psalidodon paranae* (Terán et al., 2020). These results indicated that the taxonomic status of the family Characidae is currently unresolved, and morphological classification combined with the usage of mitogenomes and other molecular markers are needed for comprehensive classification (Liu et al., 2020). These findings provide a phylogenetic basis for improved classification of the family Characidae. The newly sequenced mitogenomes of the two species (*H. erythrozonus* and *H. amandae*) improve our understanding of the phylogeny and evolution of fish species.

## Data Availability Statement

The data presented in this study can be found in GenBank with accession numbers MT484070 and MT484069.

## Ethics Statement

The animal study was reviewed and approved by the Ethics Committee of the Nanjing Forestry University.

## Author Contributions

H-YL, B-PH, and C-HS contributed to the experimental design. NX and X-LZ were involved in the sample collection and pre-processing. C-HS contributed to the data analysis and image editing. H-YL and C-HS drafted the manuscript. B-PH, QZ, and C-HS reviewed and edited the manuscript. All authors contributed to the article and approved the submitted version.

## Conflict of Interest

The authors declare that the research was conducted in the absence of any commercial or financial relationships that could be construed as a potential conflict of interest.

## Footnotes

1 http://mitos2.bioinf.uni-leipzig.de/index.py

2 https://chlorobox.mpimp-golm.mpg.de/OGDraw.html

## Supplementary Material

Supplementary Figure 1 Phylogenetic tree of 24 Actinopterygii species constructed by the Bayesian inference methods based on the concatenated sequences of 13 PCGs and two rRNAs. The support values are Bayesian posterior probabilities.

Supplementary Figure 2 Phylogenetic tree of 24 Actinopterygii species constructed by the Maximum likelihood methods based on the concatenated sequences of 13 PCGs and two rRNAs. The support values are bootstrap support values.
