# Supplementary material for: Mitochondrial Genome Structures and Phylogenetic Analyses of Two Tropical Characidae Fishes
Source: Front Genet. 2021 Feb 1;12:627402. doi: 10.3389/fgene.2021.627402 (PMC7901900; doi:10.3389/fgene.2021.627402)
Supplement: Supplementary Figure 1 — Phylogenetic tree of 24 Actinopterygii species constructed by the Bayesian inference methods based on the concatenated sequences of 13 PCGs and two rRNAs. The support values are Bayesian posterior probabilities. [file Data_Sheet_1.docx]

Supplementary Material


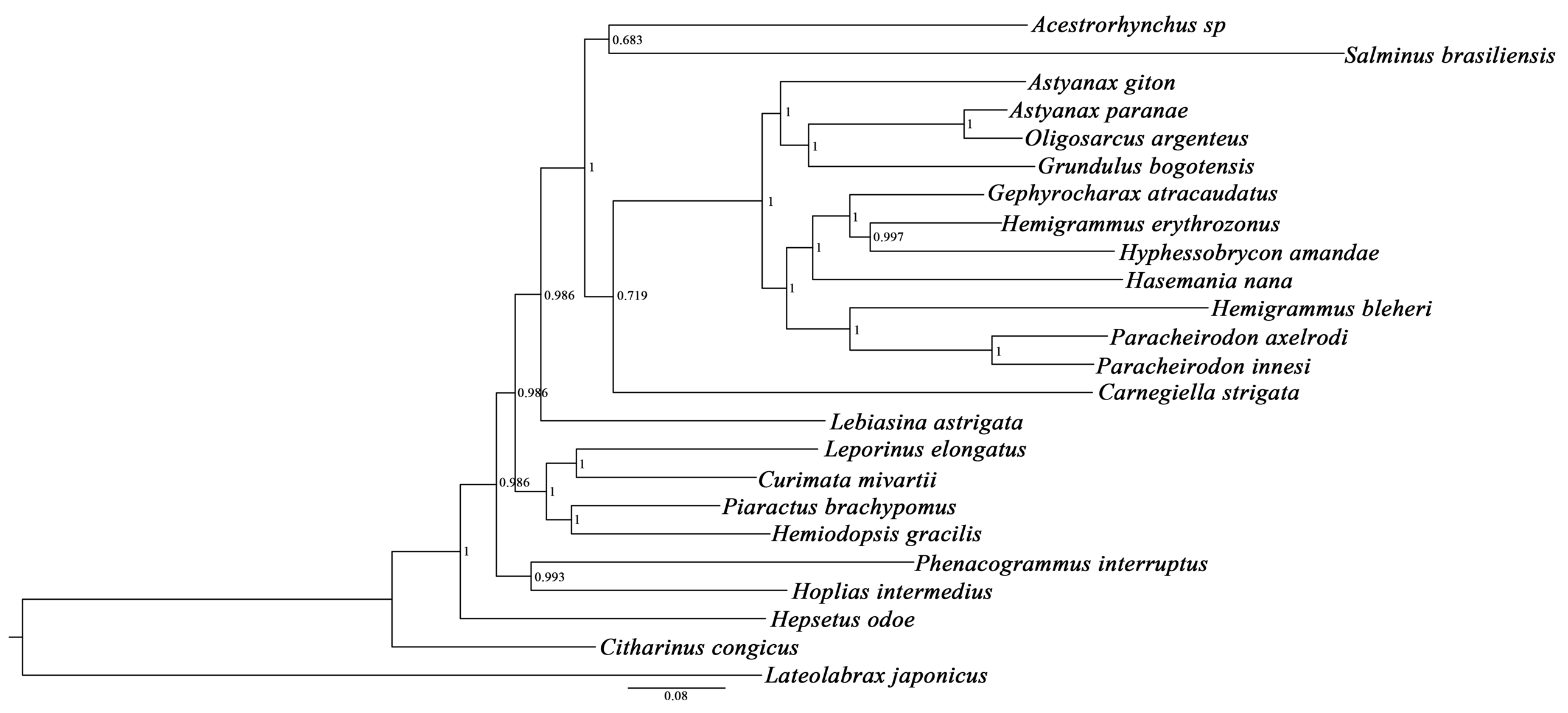


**Supplementary Figure 1.** Phylogenetic tree of 24 Actinopterygii species constructed by the Bayesian inference methods based on the concatenated sequences of 13 PCGs and two rRNAs. The support values are bayesian posterior probabilities.


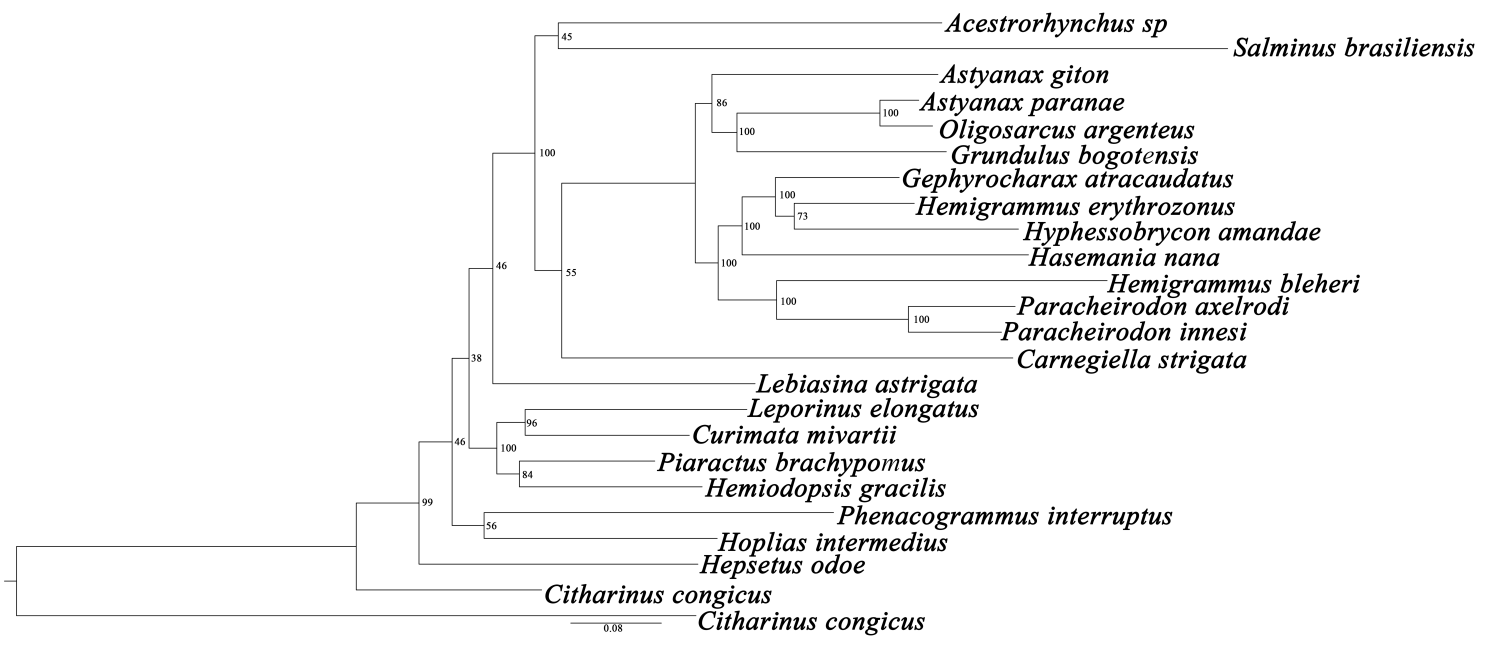


**Supplementary Figure 2.** Phylogenetic tree of 24 Actinopterygii species constructed by the Maximum likelihood methods based on the concatenated sequences of 13 PCGs and two rRNAs. The support values are bootstrap support values.
